# The Role of GM-CSF Autoantibodies in Infection and Autoimmune Pulmonary Alveolar Proteinosis: A Concise Review

**DOI:** 10.3389/fimmu.2021.752856

**Published:** 2021-11-22

**Authors:** Ali Ataya, Vijaya Knight, Brenna C. Carey, Elinor Lee, Elizabeth J. Tarling, Tisha Wang

**Affiliations:** ^1^ Division of Pulmonary, Critical Care and Sleep Medicine, University of Florida, Gainesville, FL, United States; ^2^ Department of Pediatrics, Section of Allergy and Immunology, University of Colorado School of Medicine and Children’s Hospital, Aurora, CO, United States; ^3^ Translational Pulmonary Science Center, Cincinnati Children’s Hospital Medical Center, Cincinnati, OH, United States; ^4^ Division of Pulmonary, Critical Care, and Sleep Medicine, David Geffen School of Medicine at University of California, Los Angeles (UCLA), Los Angeles, CA, United States; ^5^ Department of Medicine, Division of Cardiology, David Geffen School of Medicine at University of California, Los Angeles (UCLA), Los Angeles, CA, United States

**Keywords:** autoantibodies, autoimmune pulmonary alveolar proteinosis, sargramostim, *Nocardia*, *Cryptococcus*, *Aspergillus*, *Histoplasma*, granulocyte-macrophage colony-stimulating factor (GM-CSF)

## Abstract

Autoantibodies to multiple cytokines have been identified and some, including antibodies against granulocyte-macrophage colony-stimulating factor (GM-CSF), have been associated with increased susceptibility to infection. High levels of GM-CSF autoantibodies that neutralize signaling cause autoimmune pulmonary alveolar proteinosis (aPAP), an ultrarare autoimmune disease characterized by accumulation of excess surfactant in the alveoli, leading to pulmonary insufficiency. Defective GM-CSF signaling leads to functional deficits in multiple cell types, including macrophages and neutrophils, with impaired phagocytosis and host immune responses against pulmonary and systemic infections. In this article, we review the role of GM-CSF in aPAP pathogenesis and pulmonary homeostasis along with the increased incidence of infections (particularly opportunistic infections). Therefore, recombinant human GM-CSF products may have potential for treatment of aPAP and possibly other infectious and pulmonary diseases due to its pleotropic immunomodulatory actions.

## Introduction

Autoantibodies (AAbs) against cytokines are detectable in the plasma of both healthy and diseased individuals, but the role and potential pathology of anti-cytokine autoantibodies are not fully known (1). Cytokine-specific AAbs are considered naturally occurring immune regulators as well as pathogenic instigators. AAbs to multiple cytokines have been identified and some, including antibodies against granulocyte-macrophage colony-stimulating factor (GM-CSF), have been associated with increased susceptibility to infection. High levels of AAbs that neutralize GM-CSF signaling cause autoimmune pulmonary alveolar proteinosis (aPAP), an ultrarare autoimmune disease characterized by accumulation of excess surfactant in the alveoli, leading to pulmonary insufficiency. This review article summarizes the role of GM-CSF in aPAP pathogenesis and pulmonary homeostasis along with the increased incidence of infections (particularly opportunistic infections).

## Anti-Cytokine Autoantibodies Associated Primarily With Infectious Manifestations

### Overview of Anti-Cytokine Autoantibodies

Inborn errors of immunity involving mutations affecting selected cytokines or their signaling pathways can increase susceptibility to infections. Anti-cytokine AAbs can produce the same effects as pathogenic gene mutations or variants causing inborn errors of immunity, a phenomenon known as “autoimmune phenocopies of primary immunodeficiencies” (2). To date, associations of anti-cytokine antibodies and infections have been identified for antibodies against GM-CSF, multiple interleukins, granulocyte colony-stimulating factor (G-CSF), tumor necrosis factor-alpha (TNF-α), and various types of interferon (3–13).

AAbs are usually found complexed with cytokines, and in some (but not all) cases these AAbs are associated with cytokine-neutralizing activity (1, 8, 14). Their exact function is unclear, but they might serve to negatively regulate or neutralize cytokine activity through sequestration and subsequent degradation of autoantibody–cytokine complexes (8, 15). Such complexes could still confer biological activity *via* Fc-mediated effects, assuming the autoantibodies do not interfere with the cytokine’s biological activity (15). Alternatively, these complexes might exert agonistic effects by serving as cytokine reservoirs, thus prolonging their half-life.

### Pathogenesis of Anti-Cytokine Autoantibodies

Factors driving development of anti-cytokine AAbs in healthy individuals are not clearly understood. Exposure to *Aspergillus* can produce AAbs that cross-react with IFN-γ (due to shared epitopes) and neutralize IFN-γ function, suggesting that molecular mimicry may play a role in the generation of these antibodies (16). Patients with high levels of neutralizing IFN-γ AAbs are, therefore, at increased risk of mycobacterial infection, which is severe in the majority of cases; such patients may also be at risk for severe *Salmonella* and varicella zoster infections. Neutralizing anti–IFN-α and anti–IFN-ω antibodies also have been detected in patients with hypomorphic recombination-activating genes (*RAG*) mutations, which could inhibit the antiviral immune response and predispose patients to infections and infection-related complications (17). More recently, AAbs against IFN-α and IFN-ω have been found to be associated with cases of severe COVID-19 (18). Similarly, AAbs to IL-6 can increase the risk of severe pyogenic infections (2). IL-6 AAbs have been reported in a patient with recurrent staphylococcal cellulitis, suggesting impaired IL-6–mediated immunity may have contributed to staphylococcal disease (19). Patients with primary immunodeficiency autoimmune polyendocrine syndrome type I (APS-I) suffer from chronic mucocutaneous candidiasis (CMC) and are shown to have increased serum titers of AAbs to IL−17A/F and IL−22 (10). Defects in these signaling pathways may impair immunity and contribute to development of CMC in these patients.

### Anti-Cytokine Autoantibodies in Other Disease States

Anti-cytokine AAbs have been described for many other autoimmune diseases including rheumatoid arthritis, systemic sclerosis, multiple sclerosis, Felty’s syndrome, systemic lupus erythematosus, and sarcoidosis; however, their frequency and role have been extensively debated (20, 21). Recently, AAbs to various cytokines, including GM-CSF, were identified in some patients with postherpetic neuralgia, which were hypothesized to cause an autoimmune immunodeficiency syndrome leading to uncontrolled varicella zoster virus reactivation and nerve damage (22). While the precise role of anti-cytokine AAbs in these diseases is less clear, the presence of GM-CSF AAbs in aPAP and their role in its pathogenesis have been firmly established.

## Autoimmune Pulmonary Alveolar Proteinosis

Autoimmune PAP (previously known as idiopathic PAP) accounts for approximately 90% of all PAP cases (23). The remainder consist of secondary PAP, which is associated with various disorders (e.g., chronic inflammatory syndromes, immunodeficiencies, certain hematological diseases and solid tumors, immunoglobulin A deficiency); hereditary PAP that arises from mutations in genes for *CSF2RA* and *CSF2RB*; and congenital PAP, associated with mutations in genes for surfactant proteins (*SFTPA*, *SFTPB*, *SFTPC*), ATP-binding cassette subfamily A member 3 (*ABCA3*), and thyroid transcription factor-1 (*TTF1*).

Autoimmune PAP is characterized by high concentrations of GM-CSF AAbs that bind to GM-CSF, decreasing its level and biological activity (24). Defective GM-CSF signaling leads to functional deficits in multiple cell types, including macrophages and neutrophils, with impaired phagocytosis and cytokine production (25, 26). GM-CSF regulates the maturation, differentiation, and proliferation of monocytes and macrophages, as well as clearance of surfactant from alveolar spaces (25, 27, 28). Loss of GM-CSF signaling, as seen in aPAP and in mice functionally deficient in GM-CSF, interferes with terminal differentiation and function of alveolar macrophages (11, 27, 29, 30). This loss of alveolar macrophage functional activity leads to reduced clearance of lung surfactant, ineffective host immune defense, and respiratory failure, all of which can facilitate the proliferation and dissemination of extrapulmonary pathogens. Surfactant accumulation in PAP was shown to arise from reduced catabolism in alveolar macrophages and not increased surfactant production; surfactant metabolism in type II alveolar epithelial cells (AEC-II) was not affected (31, 32).

High-resolution CT imaging of the lungs shows diffuse bilateral alveolar infiltrates, with patchy ground-glass opacities in a characteristic “crazy paving” pattern (24). Bronchoalveolar lavage fluid is often thick and milky-appearing with granular periodic acid-Schiff–positive acidophilic acellular material, large foamy alveolar macrophages containing phospholipoprotein, and phagocytosomes. Patients with aPAP — even those who are otherwise immunocompetent — are known to be at increased risk of infection, especially due to opportunistic intracellular microorganisms. Infections can precede or follow development of aPAP clinical symptoms, or occur concurrently (33). However, the precise role and interactions between infections and aPAP are not yet fully understood.

Increased systemic GM-CSF AAbs as well as localized (pulmonary) GM-CSF AAbs are found in all patients with aPAP. Elevated serum GM-CSF AAb levels diagnostic of aPAP are considered sufficient to effectively neutralize all GM-CSF activity, suggesting that aPAP is functionally equivalent to knockout mice deficient in GM-CSF production (*Csf2^-/-^*) (34, 35). Low levels or weak neutralizing activity of GM-CSF AAbs may be sufficient to allow for partial alveolar macrophage function in some patients, whereas high-titer AAbs suppress these functions and lead to development of aPAP (2).

## GM-CSF

### Role of GM-CSF in Host Defense

**Figure 1:**
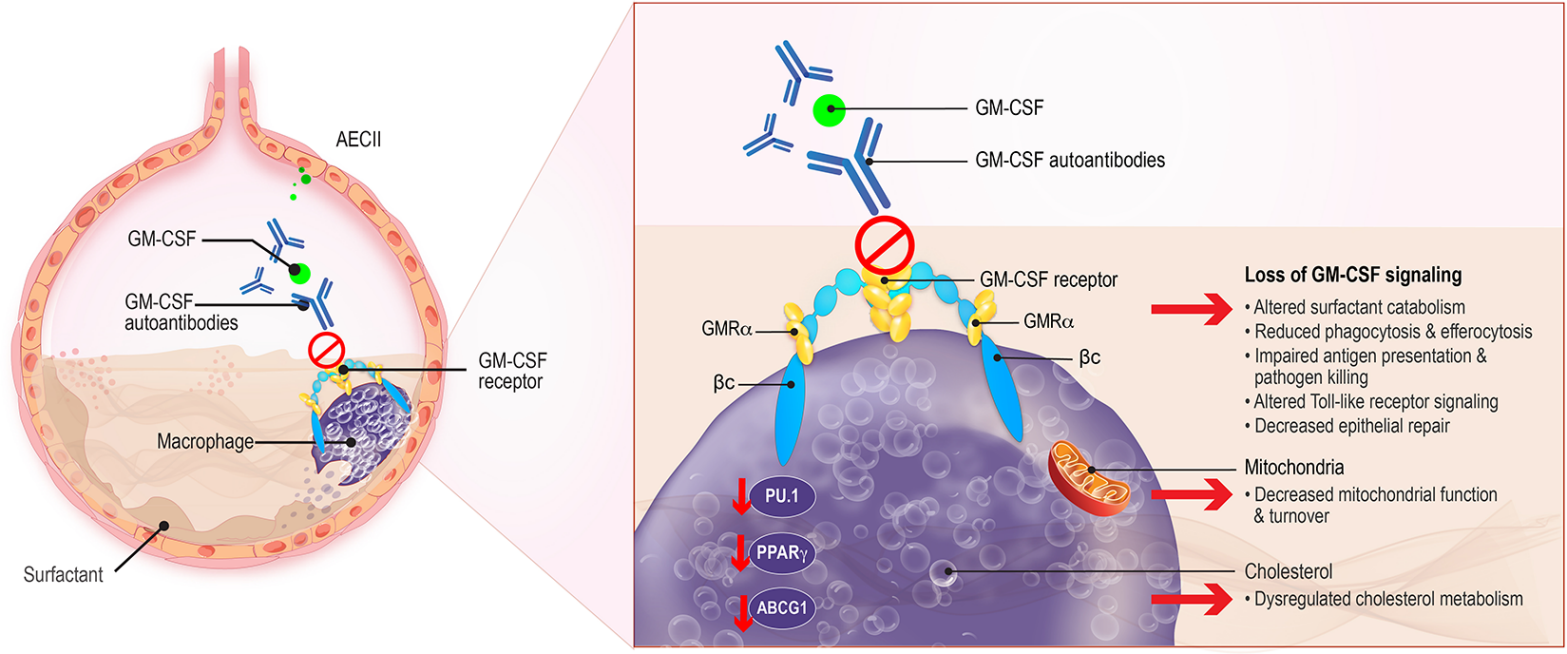
Macrophage Dysregulation in aPAP. GM-CSF, produced by alveolar epithelial cells type II (AECII) which binds to the GM-CSF receptor on AECII and macrophages, contributes to surfactant homeostasis and host defense against pulmonary microbial infections. Surfactant production is normally balanced by its catabolism by alveolar macrophages. Uptake and efflux of pulmonary surfactant from alveolar macrophages is normally regulated by GM-CSF. The presence of GM-CSF autoantibodies in aPAP interferes with GM-CSF regulation of surfactant, leading to its accumulation in alveoli. Other critical macrophage functions such as antigen presentation, phagocytosis, and Toll-like receptor signaling, are also affected. Additionally, key regulators of phospholipid metabolism such as the transcription factor PU.1 and ABCG1 are modified, leading to altered fatty acid and phospholipid catabolism and changes in amino acid biosynthesis. βc, GM-CSF receptor common β-subunit; GMRα, GM-CSF receptor subunit-α.

GM-CSF is an immunomodulatory cytokine produced by multiple cell types including macrophages, lymphocytes, endothelial cells, fibroblasts, and alveolar epithelial cells. It acts to stimulate the proliferation and differentiation of hematopoietic stem cells into granulocytes and macrophages, including the terminal differentiation of alveolar macrophages, and to promote the growth, activation, and survival of myeloid cells (36). GM-CSF also enhances neutrophil function and increases their adherence, phagocytosis, and bactericidal activity (26). In the lungs, GM-CSF is produced by AEC-II and binds to the heterodimeric GM-CSF receptor (GM-CSFR) expressed on AEC-II and alveolar macrophages (**Figure 1**) (37, 38). GM-CSF acts by initially binding to the GM-CSF receptor alpha (α) chain and recruiting the GM-CSF receptor beta (β) chain to trigger intracellular signaling, including downstream phosphorylation and activation of signal transducer and activator of transcription 5 (STAT5), and expression of transcription factors including PU.1 and peroxisome proliferator-activated receptor-γ (PPARγ) (**Table 1**) (25, 30, 37, 38). GM-CSF also modulates lipid homeostasis, increasing ABCG1 expression and inducing efflux of cholesterol from alveolar macrophages and its subsequent catabolism (56, 57).

**Table 1:** Effects of GM-CSF deficiency on alveolar macrophages in autoimmune pulmonary alveolar proteinosis.

Affected function or cellular effect	Specific alterations
Reduced host defense (39–42)	Increased susceptibility to *Streptococcus*, Pseudomonas, *Pneumocystis*, malaria, and adenoviral infection
Impaired terminal differentiation (30, 43, 44)	Altered expression of macrophage differentiation genes: BM8^neg^, Ly-6C^pos^, PU.1^reduced^
Reduced innate immune functions (25, 29, 30, 45–55)	Reduced antigen presentation Reduced microbicidal activity Reduced leukocyte chemotaxis Reduced cell adhesion Reduced phagocytosis Reduced pathogen recognition receptor expression Reduced pathogen killing Reduced LPS- or peptidoglycan-stimulated TNFα release Reduced FcγR expression: CD64^neg^, CD32^neg^, CD16^neg^ Altered integrin expression: CD11a^neg^, CD11b^pos^, CD11c^neg^ Increased pulmonary levels of M-CSF and MCP-1 Reduced STAT5 signaling
Altered lipid homeostasis (56–58)	Reduced PPARγ and ABCG1 expression Reduced cholesterol efflux Lipid accumulation
Altered mitochondrial functions (59)	Mitochondrial integrity and turnover Amino acid and nucleotide biosynthesis Cellular metabolism (tricarboxylic acid cycle, glycolysis, and pentose phosphate pathways) Energy production Redox state

LPS, lipopolysaccharide; M-CSF, monocyte colony-stimulating factor; MCP-1, monocyte chemoattractant protein-1; PPARγ, peroxisome proliferator-activated receptor gamma; STAT5, signal transducer and activator of transcription 5; TNFα, tumor necrosis factor alpha.

The effects of GM-CSF on macrophage function are extensive and include enhancing macrophage antigen presentation capacity and antibody-mediated phagocytosis *via* complement (29, 60). Macrophage microbicidal capacity, as well as leukocyte chemotaxis and adhesion, are also enhanced. GM-CSF induces the production of multiple cytokines including IL-6, IL-12p70, IL-23, and TNF-α (61, 62). Recent data from studies of GM-CSFR β-chain–deficient (*Csf2rb^–/–^*) mice suggest GM-CSF is essential for mitochondrial function and turnover, including fatty acid β-oxidation (59). GM-CSF also stimulates ATP production, tricarboxylic acid cycle activity, oxidative phosphorylation, and regulates other critical metabolic functions, thus helping to maintain cellular metabolism and cell growth (59).

**Figure 2:**
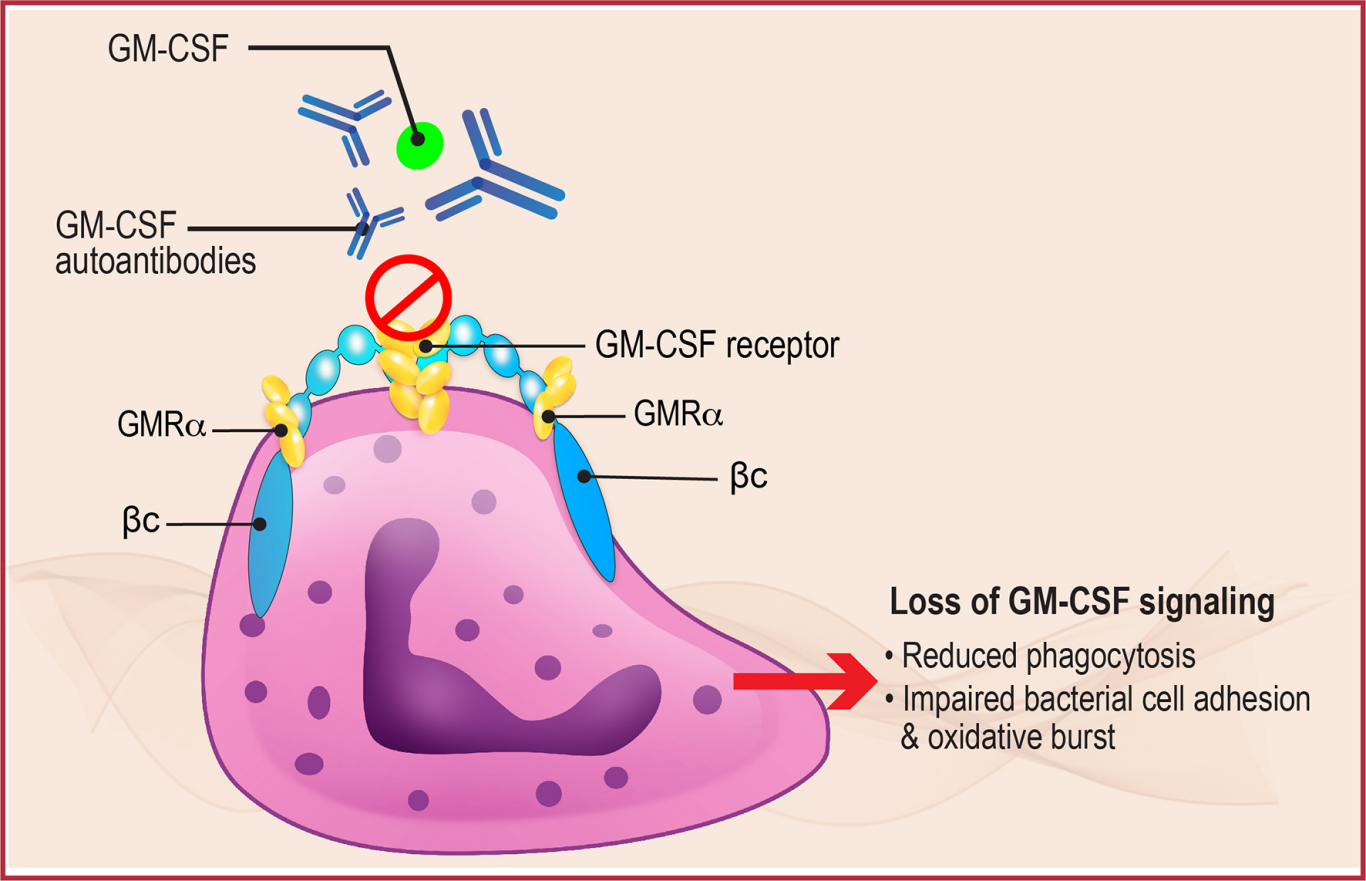
Neutrophil Dysregulation in aPAP. GM-CSF normally binds to the GM-CSF receptor, present on the surface of neutrophils (shown here) and alveolar macrophages, to initiate downstream signaling that regulates multiple functions including phagocytosis, bacterial cell adhesion, and oxidative burst. In aPAP, high levels of GM-CSF autoantibodies bind to GM-CSF preventing binding and receptor activation, thus inhibiting receptor signaling and leading to neutrophil and macrophage dysfunction. βc, GM-CSF receptor common β-subunit; GMRα, GM-CSF receptor subunit-α.

Given the key role of neutrophils in phagocytosis and intracellular killing of a variety of pathogens (**Figure 2**), inhibition of neutrophil function due to decreased GM-CSF activity could also have a significant impact on local and systemic infections (26). Neutrophils from patients with aPAP demonstrate reduced baseline functions including phagocytic ability, bacterial cell adhesion, and oxidative burst compared with normal controls, as well as reduced GM-CSF priming. This neutrophil inhibition was reproduced *in vitro* by incubating normal neutrophils with GM-CSF AAbs, and by injection of GM-CSF antibodies into wild-type mice (26). These results support the pivotal role of GM-CSF in regulating antimicrobial functions of neutrophils and explain, in part, its effects on elimination of intracellular pathogens. The results also suggest the potential for studying GM-CSF therapy in the treatment of serious infections associated with aPAP.

Damiani et al. have detailed in a separate review multiple host GM-CSF–driven functions that counter intracellular pathogens (45). For example, GM-CSF–mediated zinc (Zn) sequestration *via* metallothioneins has been demonstrated *in vitro* and *in vivo*, in mice and human macrophages, to drive phagocyte antimicrobial effector function (63). GM-CSF is also known to drive macrophage polarization. Infections trigger a complex sequence of inflammatory and immune events that vary according to type of pathogen, immune cell involvement, cytokine production, and time to resolution. Intracellular pathogens and bacterial proteins such as lipopolysaccharide can induce production of GM-CSF and IFN-γ to drive M1 macrophage polarization (46, 62). Such cells, which respond mainly during the early stages of infection, are responsible for production of chemokines and proinflammatory cytokines contributing to the differentiation and activation of T helper cells that aid in fighting infections. In contrast, certain parasitic, fungal, and helminthic infections cause M2 polarization, which are more anti-inflammatory and function primarily in tissue remodeling and homeostasis.

### Role of GM-CSF in Pulmonary Homeostasis

Alveolar macrophages and alveolar epithelial cells work in tandem to respond to insults. GM-CSF helps to maintain the alveolar epithelium and pulmonary immune system under both physiological and pathological conditions, including infection.

Early preclinical and translational studies demonstrated the pivotal role of GM-CSF in the regulation of pulmonary surfactant homeostasis and alveolar macrophage immune function. Mice deficient in GM-CSF (*Csf2^-/-^*) were generated *via* targeted ablation of the *GM-CSF* gene (32, 64). The absence of GM-CSF resulted in impaired alveolar macrophage function, surfactant accumulation, and lung histopathology similar to PAP. This pathology could be reversed by pulmonary administration of exogenous GM-CSF, adenoviral expression of GM-CSF, or retroviral vector expression of PU.1 transcription factor (25, 65, 66). Overexpression of a GM-CSF transgene in the lung epithelium of *Csf2^-/-^* mice restores normal lung histology and function as well as pulmonary surfactant homeostasis (31). To generate mice that overexpress GM-CSF in the lung, a construct of the human surfactant protein C promoter directing expression of mouse GM-CSF cDNA was injected into ova fertilized with sperm from *Csf2^-/-^* mice. Offspring were backcrossed to *Csf2^-/-^* mice (64). Alveolar macrophages from patients with aPAP were determined not to be deficient in either the production of GM-CSF or GM-CSFR function; however, they have a decreased bioavailability of GM-CSF due to the presence of GM-CSF AAbs and negative regulation by IL-10 (67, 68).

Under certain circumstances, chronic overexpression of pulmonary GM-CSF may result in adverse effects. Exposure to cigarette smoke, which increases the risk of pulmonary diseases such as lung cancer, pulmonary emphysema, and desquamative interstitial pneumonia (DIP), has been shown to stimulate pulmonary GM-CSF expression and to increase the number of alveolar macrophages or alter their function (69–72). Using a transgenic mouse model, chronic overexpression of pulmonary GM-CSF led to spontaneous activation and progressive accumulation of alveolar macrophages, increased metalloprotease expression, and parenchymal lung damage, resulting in development of emphysema, secondary polycythemia, and increased mortality (73). Chronic pulmonary GM-CSF expression was thus able to reproduce the features of DIP (74, 75). Of note, wild-type mice subjected to chronic cigarette smoke also showed increased pulmonary GM-CSF and alveolar macrophage accumulation, although these studies used high-level smoke exposure for a prolonged duration. These data suggest chronic, continuous enhanced GM-CSF expression might play a role in the pathogenesis of smoking-related DIP. GM-CSF may act more like a pulmonary hormone to regulate alveolar macrophage number and function, and chronic hyperactivation could result in progressive lung damage and onset of DIP (73).

*Csf2^-/-^* mice have been invaluable in deciphering the pathogenesis underlying the development of aPAP. Such mice recapitulate the pathological findings in PAP, have defective macrophage and neutrophil function, and exhibit compromised clearance of surfactant. As a result, *Csf2^-/-^* mice show increased susceptibility to a variety of pulmonary and systemic infections including streptococcal, *Pseudomonas aeruginosa*, *Pneumocystis carinii*, malaria, and adenovirus infections (39–41, 76). The deficiency in GM-CSF activity, leading to decreased clearance of surfactant and reduced immune function, provides a mechanistic basis for the increased rates of infection and infection-related mortality frequently seen in patients with aPAP (42).

Loss of functional GM-CSF in *Csf2^-/-^* mice and patients with aPAP is also associated with altered lipid homeostasis, including dysregulated cholesterol update and efflux. This is due in part to a deficiency in PPARγ and ABCG1 activity that regulate lipid metabolism, as well as decreased expression of the master transcription factor PU.1 that reduces surfactant catabolism (30, 56, 77). Together, these result in an accumulation of lipids within alveolar macrophages and build-up of lipid-laden pulmonary surfactant. In both *GM-CSF* and *PPARγ* knockout animal models and in patients with aPAP, extracellular deposition of lipids in pulmonary alveoli and bronchoalveolar lavage fluid can be seen (29, 77). This extracellular lipid accumulation could lead to decreased access to and function of immune cells, further contributing to the persistence and growth of infections and slowing the removal of pathogens and cellular debris.

## Detection and Functional Evaluation of GM-CSF Alterations in aPAP

### Clinical Assessment of GM-CSF Autoantibodies

Clinical lab evaluation of serum GM-CSF AAbs is routinely performed using a standardized ELISA, with a reported sensitivity and specificity of 100% for the diagnosis of aPAP (35). GM-CSF AAb levels are much higher in the sera of patients with aPAP compared with patients who have other pulmonary diseases or healthy controls (58). The presence of GM-CSF AAbs is instrumental in confirming the diagnosis of aPAP; however, the presence of pulmonary manifestations is necessary for a clinical diagnosis. Asymptomatic individuals with elevated GM-CSF AAbs may potentially be at risk of developing the clinical manifestations and complications of aPAP.

GM-CSF AAbs can be detected in patients with cancer and selected inflammatory conditions, albeit at low levels (15, 35, 78). GM-CSF AAbs also are measurable in healthy individuals but at levels 10- to 100-fold less than that observed in aPAP. Such antibodies in healthy individuals are usually found to be complexed with GM-CSF and thus are biologically less active. Routine clinical testing for GM-CSF AAbs is available only through specialized laboratories in the United States (Cincinnati Children’s Hospital Medical Center, National Jewish Health), Japan, Germany, and China (more information regarding testing is available from the PAP Foundation and EuPAPNet) (79). It should be emphasized that although diagnostic for aPAP, GM-CSF AAb levels do not reliably correlate with disease severity (15, 35, 78).

A study of patients with aPAP found that GM-CSF AAb levels were significantly lower at baseline or gradually declined over time in patients who spontaneously improved compared with those who did not (58). Decreases in GM-CSF AAb levels in serum and/or bronchoalveolar lavage fluid correlate with clinical response to aPAP therapy in some studies (24, 80–82) but not in others (83, 84). The reasons for these disparate findings are not fully understood but could be due in part to intra-patient variation or differences in AAb quantitation methodology. These results, if confirmed in larger studies, suggest that levels of serum GM-CSF AAbs might reflect disease persistence for individual patients even if they are not prognostic across the entire aPAP population. Further research is needed to better understand the role of GM-CSF AAb levels in the aPAP patient population.

In addition to standardized ELISA assays, AAbs to GM-CSF, IFN-γ, IL-2, and other cytokines have been detected using alternative techniques such as bead multiplex assay (17); however, such research-based approaches are generally not used for routine clinical diagnosis. Similarly, novel approaches such as immune-mass cytometry are being explored but not yet developed for detection of GM-CSF AAbs.

### Measurement of GM-CSF Serum Levels and GM-CSF Function

**Figure 3:**
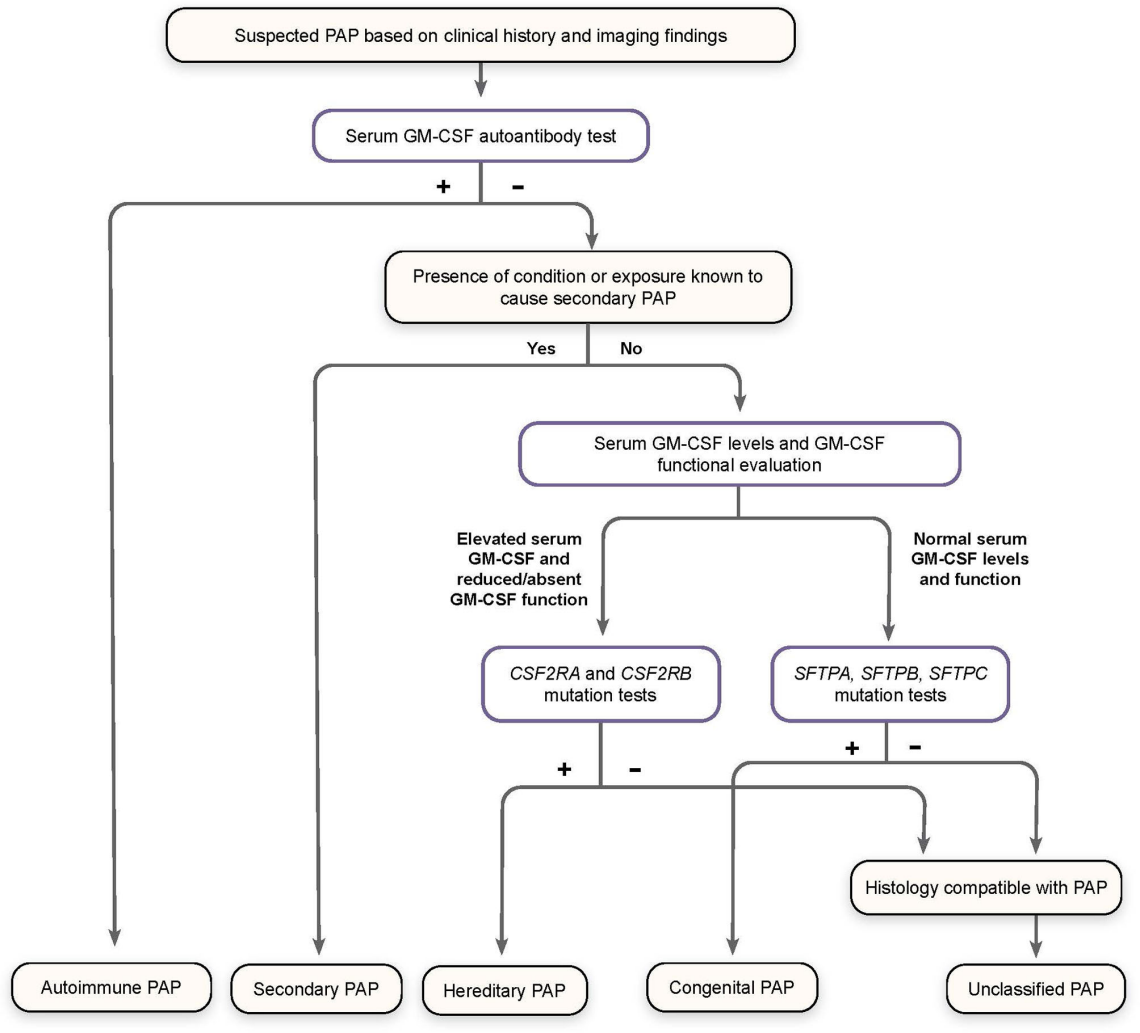
Algorithm for Diagnosis of Pulmonary Alveolar Proteinosis (PAP). Patients suspected of having PAP on the basis of clinical history and imaging findings should undergo screening for serum GM-CSF autoantibodies. Elevated autoantibody levels confirm a diagnosis of autoimmune PAP. Patients with a known condition or exposure associated with secondary PAP who do not have increased GM-CSF autoantibodies are diagnosed accordingly. Otherwise, patients should be screened for serum GM-CSF levels and GM-CSF function signaling by flow cytometry, as well as genomic analysis for mutations affecting GM-CSF receptor genes (*CSF2RA* and *CSF2RB)* and those related to surfactant production (*SFTPA, SFTPB, SFTPC)*, to confirm a diagnosis of hereditary, congenital, or unclassified PAP, all of which are exceedingly rare. (Adapted from “Pulmonary alveolar proteinosis,” by BC Trapnell, 2019, *Nat Rev Dis Primers, 5:16:8.*).

Patients with symptoms of PAP but a low level or absence of GM-CSF AAbs (effectively excluding the diagnosis of aPAP) should be assessed for GM-CSF functional signaling and serum GM-CSF concentration (**Figure 3**) (79).

Alterations in GM-CSF signaling can be detected by flow cytometry functional assays based on the ability of GM-CSF to stimulate cell-surface CD11b expression and intracellular STAT5 phosphorylation. The resulting CD11b stimulation index can effectively discriminate between healthy individuals and those with aPAP who exhibit significantly reduced induction of cell surface CD11b expression. This method has a reported sensitivity and specificity of 100%, aiding in the diagnosis of aPAP (85). Quantification of intracellular phosphorylated STAT5 (pSTAT5) in whole blood or peripheral blood mononuclear cells can similarly discriminate between healthy individuals and those with aPAP (8). Routine clinical testing for GM-CSF functional signaling is available only through specialized laboratories in the United States (Cincinnati Children’s Hospital Medical Center and National Jewish Health) (**Table 2**). In the future, functional assays may also have potential for therapeutic monitoring and to inform treatment decisions.

**Table 2:** Clinical diagnostic testing for pulmonary alveolar proteinosis syndrome.

Clinical diagnostic test	Disease evaluated
Serum GM-CSF autoantibody level	aPAP
Serum GM-CSF level	Hereditary PAP (GM-CSFR dysfunction)
GM-CSFR signaling analysis	Primary PAP (aPAP and GM-CSFR dysfunction)
*CSF2RA* gene sequence analysis	Hereditary PAP (GM-CSFRα dysfunction)
*CSF2RB* gene sequence analysis	Hereditary PAP (GM-CSFRβ dysfunction)

aPAP, autoimmune pulmonary alveolar proteinosis; GM-CSF, granulocyte-macrophage colony-stimulating factor; GM-CSFR, granulocyte-macrophage colony-stimulating factor receptor; PAP, pulmonary alveolar proteinosis.

Serum GM-CSF should be measured for patients with normal GM-CSF AAb levels who do not have obvious causes of secondary PAP (79). Elevated serum GM-CSF concentrations (>10 pg/mL) are present in patients with hereditary PAP but are generally undetectable in normal individuals. This could be because GM-CSF induces more paracrine effects and may be limited by tissue specificity, or due to the fact that in healthy subjects more than 99% of serum GM-CSF is bound and neutralized by endogenous GM-CSF AAbs and therefore may not be detected by standard assays for GM-CSF AAb levels (47). GM-CSF levels also can be increased as a result of infection or systemic inflammation in the absence of hereditary PAP (86–89). If GM-CSF AAb levels, serum GM-CSF levels, and functional tests prove normal with no known cause of secondary PAP, genomic analysis can be used to rule out congenital PAP (79). Histopathology of lung biopsies as a last resort may confirm PAP by demonstrating the presence of granular periodic acid-Schiff–positive material and foamy alveolar macrophages, although this does not specify the etiology of the PAP and is discouraged as a routine diagnostic aid given the invasive nature of the procedure.

In summary, detection of GM-CSF AAb levels, along with functional assays and determination of serum GM-CSF levels, can confirm or rule out a suspected diagnosis of aPAP and may also aid in monitoring disease persistence.

## Infections in aPAP

### Overview of Infections in aPAP

Infections are common in patients with aPAP. Additionally, individuals with GM-CSF AAbs can develop unusual pulmonary and extrapulmonary infections at rates exceeding expected complications of their lung disease (90). The onset of PAP may occur concurrently with, or be temporally distinct from, opportunistic infections (33).

### Common Opportunistic Infections

Approximately 4% to 6% of PAP patients present with opportunistic infections, typically *Nocardia*, mycobacteria, and fungal infections (**Table 3**) (23, 33, 42). This predisposition to infection is systemic, rather than confined to the lungs. Although organisms common in community- and hospital-acquired infections have been reported in PAP (e.g., *Streptococcus*, *Klebsiella*, *Hemophilus*, *Staphylococcus*, *Pseudomonas*, *Serratia*, *Proteus*, *Escherichia*), more often reports have identified opportunistic microbial pathogens (27, 90). In a study of 212 Japanese patients with aPAP, 12 (5.7%) were found to have concurrent infections (primarily aspergillosis, atypical mycobacteria, and *Mycobacterium tuberculosis*), of which at least half were pulmonary infections (23). Superinfection with H1N1 influenza also has been reported, which can exacerbate aPAP (91). While the outcome of these infections is determined by a complex set of interacting pathogen and host factors, such infections are often associated with significant morbidity and mortality. As previously outlined, anti-cytokine antibodies including those against GM-CSF are increasingly appreciated as a cause of acquired immune dysregulation syndromes (90).

**Table 3:** Opportunistic pathogens commonly seen in patients with autoimmune pulmonary alveolar proteinosis.

**Bacterial**	
*Nocardia* spp.	
*Mycobacterium tuberculosis*	
*Mycobacterium avium intracellulare*	
*Mycobacterium kansasii*	
*Streptomyces*	
**Fungal**	
*Aspergillus* spp.	
*Cryptococcus* spp.	
*Histoplasma* spp.	
Zygomyces	
*Pneumocystis jiroveci*	

### Disruption of GM-CSF Signaling in aPAP

Data suggest the downstream effects of GM-CSF AAbs in the context of aPAP have broad immunological effects on monocytes, macrophages, and neutrophils (25, 26). Understanding the immunologic effects of GM-CSF on Toll-like receptor activation, phagocytosis, bactericidal activity, oxidative burst, and cell adhesion in neutrophils and macrophages provide a physiologic rationale for the infectious pathology associated with GM-CSF AAbs. These broad effects can be deleterious for host control of certain pathogens.

Disruption in GM-CSF signaling (due to the presence of AAbs) suppresses alveolar macrophage function and facilitates pulmonary infections. Defective alveolar macrophage function could also contribute to extrapulmonary dissemination of infections seen in aPAP. GM-CSF is known to regulate alveolar macrophage production of multiple factors involved in local and systemic response to infection including prostaglandins, leukotrienes, and oxygen radicals (43). This dysregulation of GM-CSF signaling inhibits the inflammatory response to lipopolysaccharide (LPS) associated with many pulmonary bacterial infections (92). Alveolar macrophages from *Csf2^-/-^* mice have been shown to exhibit reductions in multiple immune functions including cell adhesion, phagocytosis, toll-receptor signaling, bacterial killing, and expression of pathogen-associated molecular pattern recognition receptors, all of which can contribute to an increase in infections (29). Studies of adenovirus infection of alveolar macrophages indicate GM-CSF acts through the master transcriptional regulator PU.1 to inhibit their infection, prevent lysosomal translocation, and promote virion destruction (48). In mice, neutralization of GM-CSF by administration of anti–GM-CSF antibodies exacerbated pulmonary histoplasmosis infection, indicating GM-CSF regulates antimicrobial defenses against this and possibly other opportunistic infections (93).

### GM-CSF Autoantibodies and Disseminated Infections

The lungs are the predominant site for aPAP-related infections. However, extrapulmonary infections also can occur, in some cases concurrent with respiratory infections. In aPAP, deficient pulmonary macrophages may result in an ineffective pulmonary barrier, thereby facilitating dissemination beyond the lung (11).

Nocardiosis is an opportunistic infection that typically occurs in patients who are immunocompromised, especially those with defects in phagocytosis (94). While generally limited to pulmonary or cutaneous infections, dissemination is possible, including to the central nervous system (CNS). In a review of published studies of opportunistic infections in patients with aPAP, 61% were due to *Nocardia* (42). This pathogen induces GM-CSF–mediated STAT5 phosphorylation in monocytes, indicating that this pathway is important in host immune response to this organism (95). Conversely, the presence of neutralizing GM-CSF AAbs, which in a separate study were detected in 5 of 7 patients with disseminated/extrapulmonary CNS nocardiosis, could abrogate the effects of GM-CSF and thus promote susceptibility and dissemination of *Nocardia* and perhaps other opportunistic infections (11).

As with nocardiosis, cryptococcemia is rare for those individuals with intact immunity; however, impairment of first-line antimicrobial defense mechanisms — as seen in aPAP — may contribute to progression to disseminated disease (96). The presence of GM-CSF AAbs appears to increase risk for CNS infection with *C. gattii* although not with *C. neoformans* (97).

The occurrence of both localized and disseminated infections indicates that the immune dysfunction and predisposition to infection observed in aPAP occur at the systemic level and are not limited to only the respiratory system. In support of this, *Csf2^-/-^* mice also are predisposed to both pulmonary and extrapulmonary infections by a wide array of bacterial, fungal, and viral pathogens. These mice exhibit immunological defects that result in decreased ability to eliminate a variety of bacterial, viral, parasitic, and opportunistic infections (41, 44, 98, 99). Such mice also exhibit increased susceptibility to experimentally-induced autoimmune disorders, defects in the vascular extracellular matrix, reproductive effects, and increased mortality (100, 101). The widespread, multisystemic nature of these effects in the *Csf2^-/-^* mouse model, in which immunological defects are expressed at the whole-animal level, suggests aPAP is manifested by a state of systemic functional GM-CSF deficiency rather than simply an isolated pulmonary syndrome.

Based on the efficacy and safety seen to date and its known activation of both the innate and adaptive immune responses, rhu GM-CSF may concurrently aid in elimination of infectious pathogens by enhancing the host immune response in patients with aPAP, in addition to overcoming GM-CSF neutralization. Of note, three forms of rhu GM-CSF are described in the literature: sargramostim (yeast-derived), molgramostim (bacteria-derived), and regramostim (Chinese hamster ovary cell–derived); however, sargramostim is the only form commercially available. The efficacy of inhaled rhu GM-CSF, particularly following whole-lung lavage, indicates that this approach may serve as a useful therapy in aPAP (102–104). Preliminary data also support the potential use of long-term rhu GM-CSF inhalation therapy, with responses seen in patients with aPAP who failed to respond or relapsed following repeated whole-lung lavage procedures (103, 105–107).

While preliminary data does not seem to support that administration of inhaled rhu GM-CSF leads to an increase in GM-CSF AAb levels, current assays measure only the concentration of “free” GM-CSF AAb (i.e., AAb not complexed with circulating GM-CSF or bound to cells). If administration of exogenous GM-CSF saturates available GM-CSF AAbs, it could hinder the ability to accurately measure AAb levels. Currently, the concentration of GM-CSF needed to saturate such AAbs *in vivo* is not known, but this is an area of active investigation.

## Discussion

Patients with GM-CSF AAbs and aPAP are known to be at increased risk of infection, especially from opportunistic microorganisms. Deficiencies in the immune functions of alveolar macrophages and neutrophils, such as impaired phagocytosis and cytokine production, and increased surfactant accumulation are believed to enhance the risk of infection, even in otherwise immunocompetent patients. In addition to localized pulmonary infections, patients with GM-CSF neutralizing AAbs are also at risk for disseminated infections by opportunistic intracellular pathogens and of subsequent development of aPAP (11, 97). The precise role and interactions between infections and GM-CSF in patients with aPAP are not fully understood, however.

In some autoimmune diseases and infections, the level of certain cytokine AAbs may correlate with disease severity and progression. For those disease conditions in which levels of anti-cytokine AAbs do correlate with changes in clinical symptoms, such as lupus and rheumatoid arthritis, AAb levels might serve as surrogate markers of disease progression and/or response to therapy (20, 108, 109). This does not appear to be the case with GM-CSF AAb levels in aPAP (110), however, suggesting that differences exist in the functions and/or concentrations of individual cytokines in the pathogenesis of specific diseases. More studies are also necessary to better elucidate the role of GM-CSF AAb levels in individual patients as their disease progresses and/or improves.

Greater use of testing for GM-CSF AAbs in general has been recommended to improve the noninvasive detection and diagnosis of aPAP (111, 112). Such screening is particularly important since up to one third of patients with aPAP may be asymptomatic at diagnosis (23). GM-CSF AAbs were detected, for example, in 5 of 7 adults who had CNS nocardiosis but were otherwise healthy (11). Screening for aPAP is not routinely performed due to the low incidence of this disease, but AAb assessment may be considered in selected patients with abnormal chest radiology results and unusual infections. Wider testing for GM-CSF AAbs might also allow for monitoring of patients who are at increased risk of developing aPAP and other immunodeficiencies.

It should be noted that diagnosis of aPAP takes several months, often 18 months or longer after initiation of symptoms (111). Part of this lag in diagnosis could stem from the delay of an aPAP evaluation following diagnosis and treatment of a classically-associated opportunistic infection such as *Nocardia*, especially in the absence of other apparent comorbidities. Awareness of this lag could provide additional rationale for clinicians to evaluate GM-CSF AAb levels in all patients with opportunistic infections who otherwise appear immunocompetent to confirm or rule out an underlying diagnosis of aPAP.

Given the increased risk of infections (particularly opportunistic infections) with aPAP, it is imperative that such patients be monitored for possible infections and infectious complications. Routine preventive vaccinations for influenza and pneumococcal infection are also important (113). Although standardized clinical practice guidelines for PAP are lacking, the increased susceptibility to organisms such as *Pneumocystis jiroveci* and *Nocardia* spp. suggests that trimethoprim/sulfamethoxazole prophylaxis could be considered for all patients with significant disease (24). Such prophylaxis has been used successfully by other investigators for patients with nocardiosis and concurrent aPAP or primary immunodeficiency (6, 11, 114).

Patients with certain chronic autoimmune disorders such as rheumatoid arthritis and psoriasis can have increased levels of GM-CSF in serum and affected tissues, suggesting a possible role for this cytokine in the pathogenesis of these conditions and providing the rationale for therapeutic targeting of GM-CSF using exogenous GM-CSF monoclonal antibodies (115, 116). The role of GM-CSF in the immune response against aPAP-associated infections raises the question, however, of why more opportunistic infections are not seen in patients who receive anti–GM-CSF therapy in patients with rheumatoid arthritis and psoriasis. Results from preclinical studies suggest a possible explanation. *In vitro*, at least three non–cross-competing antibodies are required to generate stable immune complexes with GM-CSF and trigger their Fc-dependent degradation (34). Treatment using a single GM-CSF monoclonal antibody would not be sufficient to induce immune complex formation and subsequent degradation of GM-CSF that could increase risk of infection.

This review highlights opportunities for enhanced awareness of testing for GM-CSF AAbs in immunocompetent individuals with certain infections. More research is needed on the intrapatient correlation of GM-CSF AAb levels and severity of disease to help guide treatment. Further study is warranted on optimal treatment strategies and the potential use of sargramostim in patients with high GM-CSF AAb levels, aPAP, and concurrent infection.

## Author Contributions

All authors contributed to co-development of the manuscript including conceptualization, selection of content for inclusion, and review, editing, and revising of content. All authors approved submission for publication and accept accountability for the content.

## Funding

This work was supported by Partner Therapeutics, Inc.

## Conflict of Interest

AA serves on the Medical and Scientific Advisory Board of the PAP Foundation. VK received support for attending workshops, meetings, and education in leadership roles for Workshop in Primary Immunodeficiencies, College of American Pathologists, and Clinical Immunology Society, and served as President/Past President of the Association of Medical Laboratory Immunologists. BCC’s institution received an NIH-funded R01 grant related to the content of the manuscript. Outside of the current work, BC is a consultant to Partner Therapeutics, Inc. and serves on the Board of Directors and as Secretary/Treasurer of the PAP Foundation. EL’s institution received NIH-funded grants related to the content of the manuscript. EL has served as a consultant to Guidepoint Global and has received travel support from the PAP Foundation and the Rare Lung Disease Consortium. TW’s institution received a fellowship grant funded by Partner Therapeutics, Inc. TW received consultant fees from Partner Therapeutics for research outside of the current work; participated on an Advisory Board for IQVIA; and serves on the Board of Directors, Medical and Scientific Advisory Board, and as Vice President and Clinical Director of the PAP Foundation.

The authors declare this study received funding from Partner Therapeutics, Inc. The funder had the following involvement with the study: Professional medical writing, graphic artist, and publication fees for this manuscript.

The remaining author declares that the research was conducted in the absence of any commercial or financial relationships that could be construed as a potential conflict of interest.

## Publisher’s Note

All claims expressed in this article are solely those of the authors and do not necessarily represent those of their affiliated organizations, or those of the publisher, the editors and the reviewers. Any product that may be evaluated in this article, or claim that may be made by its manufacturer, is not guaranteed or endorsed by the publisher.
